# Early Detection of Pacing-Induced Cardiomyopathy Using MicroRNA-208b-3p and MicroRNA-9: A Prospective Cohort Analysis

**DOI:** 10.3390/genes17010103

**Published:** 2026-01-19

**Authors:** Onoufrios Malikides, Aleksi Sallo, Andria Papazachariou, Ioannis Kopidakis, Angeliki Alifragki, Joanna Kontaraki, Konstantinos Fragkiadakis, Gregory Chlouverakis, Eleftherios Kallergis, Emmanuel Simantirakis, Maria Marketou

**Affiliations:** 1 Medical School, University of Crete, 71003 Heraklion, Greece; onoufriosmalikides@gmail.com (O.M.); salloalex309@gmail.com (A.S.); apapazachariou@hotmail.com (A.P.); kopidakisg@gmail.com (I.K.); alifrangie@gmail.com (A.A.); kontarai@uoc.gr (J.K.); fragkiadakisk@hotmail.com (K.F.); gchlouve@uoc.gr (G.C.); ekallergis@med.uoc.gr (E.K.); simantir@uoc.gr (E.S.); 2 Department of Cardiology, University General Hospital of Heraklion, 71003 Heraklion, Greece; 3 Department of Internal Medicine, University General Hospital of Heraklion, 71003 Heraklion, Greece

**Keywords:** pacing-induced cardiomyopathy, global longitudinal strain, miR-208-3p, miR-9, biomarkers

## Abstract

Background/Objectives: Pacing-induced cardiomyopathy (PiCM) is a recognized complication of chronic right ventricular pacing (RVP), characterized by left ventricular (LV) dysfunction, adverse remodeling, and progression to heart failure. MicroRNAs (miRs) regulate gene expression and play an important role in ventricular remodeling. This study aimed to observe whether dynamic changes in miRs according to a novel peripheral blood mononuclear cell (PBMC)-based approach could serve as early predictive biomarkers of PiCM. Methods: A prospective, single-center cohort study was conducted in adult patients undergoing pacemaker implantation. Clinical characteristics, echocardiographic parameters and expression levels of miR-208b-3p and miR-9 were assessed immediately and 3 months post-pacemaker implantation. PiCM was defined as a ≥10% reduction in LVEF at one year, with no alternative cause. Statistical analyses included correlation testing, ROC curve analysis, and multivariate regression to identify factors associated with PiCM. Results: Among 126 patients, 11.1% developed PiCM. Compared with the non-PiCM group, those who developed PiCM exhibited more pronounced 3-month changes in miR-208b-3p (median Δ_3_log miR: +1.3 vs. −0.4, *p* = 0.013) and miR-9 (median Δ_3_log miR: −1.7 vs. +0.21, *p* = 0.011). In multivariate analyses, Δ_3_LV-GLS, Δ_3_logmiR-208b-3p, and Δ_3_logmiR-9 were associated with a higher likelihood of PiCM. Among PiCM patients, Δ_3_logmiR-208b-3p correlated inversely with Δ_3_LV-GLS (r = −0.73, *p* = 0.016), while Δ_3_logmiR-9 correlated positively (r = 0.88, *p* < 0.001). ROC analyses demonstrated good predictive ability for Δ_3_LV-GLS (AUC = 0.924), Δ_3_log miR-208b-3p (AUC = 0.783), and Δ_3_log miR-9 (AUC = 0.835), with no significant differences between curves. Conclusions: Early LV-GLS deterioration and dynamic changes in expression of miR-208b-3p and miR-9 in PBMCs precede overt LV systolic dysfunction. These miRs may serve as early predictive biomarkers for PiCM.

## 1. Introduction

Pacing-induced cardiomyopathy (PiCM) is a well-recognized complication of chronic right ventricular pacing (RVP), characterized by left ventricular (LV) dysfunction, adverse remodeling, and eventual heart failure (HF). Reported incidence rates of PiCM range from 12% to 25%, and it is linked to an increased long-term risk of HF hospitalization, atrial fibrillation (AF) and all-cause mortality [1,2]. Despite the limited evidence available on the risk factors for PiCM, previous studies have identified several clinical and pacing-related parameters associated with its development, such as male sex, baseline LV function, paced QRS duration, AF and a high percentage of RVP [3]. Yet, evidence regarding the pathophysiological mechanisms, risk factors, and early diagnostic markers of PiCM remains scarce [4,5]. Therefore, identifying high-risk individuals and establishing early diagnostic tools before the onset of systolic dysfunction represent major clinical challenges.

Recent research has highlighted the potential of left ventricular global longitudinal strain (LV-GLS) assessment as a more sensitive echocardiographic marker for detecting subtle myocardial dysfunction that precedes a decline in ejection fraction [6,7]. It has been widely applied in various clinical settings, including the evaluation of cardiomyopathies, the assessment of the impact of valvular lesions on myocardial performance, the quantification of mechanical dyssynchrony in patients at risk of ventricular arrhythmias, the diagnosis of myocardial Ischemia, and as a supplementary Index of LV filling pressure [8]. Reduced LV-GLS has also been described as a predictor of PiCM in certain studies [9]. However, the accuracy and reproducibility of LV-GLS measurements can be influenced by technical factors as well as by between-vendor variability. Therefore, the integration of complementary biomarkers, such as microRNA (miR), may provide a more objective and biologically grounded approach for identifying patients at risk of PiCM [10].

MiRs, small, non-coding RNA molecules that regulate gene expression—have been identified as potential prognostic biomarkers in several cardiovascular diseases, since their dysregulation has been implicated in myocardial fibrosis, hypertrophy, and adverse remodeling [11,12]. Among these, miR-208b-3p—a cardiac-specific miR encoded within the β-myosin heavy chain (*MYH7*) gene and involved in myocardial contractility, fibrosis, and hypertrophy—and miR-9, which modulates key signaling pathways influencing fibroblast activation and extracellular matrix deposition, have the potential to serve as prognostic biomarkers for PiCM [13]. As far as PiCM is concerned, studies investigating changes in miRs expression levels remain extremely limited in the current literature [14]. Nevertheless, recent emerging evidence further highlights the central role of miRs in cardiac remodeling and arrhythmogenesis [15,16].

The assessment of miRs expression in peripheral blood mononuclear cells (PBMCs) compared with circulation, offers additional biological relevance in chronic conditions, as PBMCs represent an active cellular interface between systemic inflammatory responses and myocardial remodeling [17]. Assessing miRs within PBMCs provides a novel and clinically relevant perspective, as recent cardiovascular literature increasingly recognizes PBMC-derived miR signatures as sensitive indicators of chronic inflammation, oxidative stress, and subclinical myocardial remodeling [17,18].

Our aim was to investigate whether dynamic changes in miR-208-3p and miR-9 expression within PBMCs could serve as predictors for the development of PiCM in PM-dependent patients.

## 2. Materials and Methods

### 2.1. Study Design and Population

This is a prospective single-center observational study in 126 adults who were sequentially admitted to the Cardiology Department of the University Hospital of Heraklion, Crete, Greece, for PM implantation for advanced heart block from September 2023 until September 2024. Inclusion criteria included patients older than 18 years who underwent PM implantation due to advanced atrioventricular (AV) block and a documented RVP burden > 40%. The exclusion criteria included baseline LVEF < 50%; known coronary artery disease (CAD) or prior myocardial infarction (MI); known cardiomyopathy, history of myocarditis; moderate or severe valvular heart disease (VHD); congenital heart disease (CHD); active malignancy or patients under chemotherapy or immunotherapy; hyperthyroidism; end-stage renal or hepatic disease; and systemic inflammatory or autoimmune diseases.

Data recorded and evaluated included age, gender, cause of PM implantation, past medical history [arterial hypertension (AH), hyperlipidemia, type 2 diabetes (T2D), HF, coronary artery disease (CAD), AF], chronic medication use [anti-hypertensive, hypolipidemic, anti-diabetic, diuretics, anticoagulants, proton pump inhibitors (PPIs)], laboratory examinations [full blood count, renal and liver function, beta-type natriuretic peptide (BNP), C-reactive protein (CRP)], echocardiographic findings [LVEF, LV-GLS, left ventricular end systolic and end diastolic diameter (LVESD and LVEDD, respectively)] and miRs (miR208b-3p and miR-9). The first assessment of all baseline clinical, laboratory, echocardiographic, and miR variables was performed immediately post-PM implantation, ensuring standardized initial measurements for all participants and avoiding the confounding effects of severe pre-implant bradycardia due to AV block on LV function. This approach allowed subsequent changes, particularly in echocardiographic and miR parameters, to reflect chronic-pacing related remodeling rather than the acute hemodynamic correction following restoration of heart rate.

Follow-up assessments were conducted during outpatient visits at 3 months and 1 year. These evaluations included a physical examination, assessment of clinical symptoms, 12-lead electrocardiography and comprehensive echocardiographic examination. RVP burden was assessed through device interrogation performed at the 3-month follow-up visit, which represents the cumulative percentage of right ventricular paced beats recorded from the time of implantation up to the 3-month assessment. In addition, a peripheral venous blood sample was obtained at the 3-month visit for a second miR analysis. All patients were prospectively followed for the development of PiCM after 1 year of follow-up, defined as a ≥10% reduction in LVEF from a baseline >50%, in the absence of alternative explanatory causes, and often accompanied by symptomatic decline [19]. This study was approved by the Ethics Committee of the University General Hospital of Heraklion, and written informed consent was obtained from all participants.

### 2.2. RNA Isolation and miR Quantification

Blood samples from all patients were collected in EDTA-coated Vacutainer tubes on the day of admission. PBMCs were isolated from 6 mL blood samples by density gradient centrifugation using Lymphoprep (Stem Cell Technologies Inc., Vancouver, BC, Canada) resuspended in TRI-Reagent (Sigma-Aldrich, St. Louis, MO, USA) and stored at −80 °C until analysis. Total RNA was isolated using the TRI-Reagent (Sigma-Aldrich Co. LLC, St. Louis, MO, USA) according to the standard protocol. RNA concentration and purity were determined using a Nanodrop spectrophotometer (Thermo Fisher Scientific™, Waltham, MA, USA). The reverse transcription of 1 μg RNA was performed using the miR-X miR First-Strand Synthesis kit (Clontech, Takara Bio Inc., Otsu, Shiga, Japan). Measurements of miR levels were performed by quantitative real-time polymerase chain reactions (qPCRs) running for 40 cycles, using the Corbett Rotor-Gene 6000 Real time PCR detection system. The KAPA SYBR FAST qPCR Kit (Kapa Biosystems, Woburn, MA, USA) was used for qPCR assays. Primers used were 5′-ATA AGA CGA ACA AAA GGT TTG T-3′ for miR-208b-3p and 5′-TCT TTG GTT ATC TAG CTG TAT GA-3′ for miR-9. The standard curve method was used for absolute quantification of the amplification products and specificity was determined by performing a melting curve analysis. U6 expression was used as a normalization standard as suggested by the miR-X miR First-Strand Synthesis kit (Clontech, Takara Bio Inc., Otsu, Shiga, Japan) using the U6 Forward Primer and the U6 Reverse Primer provided by the kit. Relative quantification of the amplification products was performed using the comparative delta-delta Ct (2-ddCt) method. All samples were run in duplicates and Ct values were averaged for the replicates.

### 2.3. Statistical Analysis

Patients were divided into two groups according to the development of PiCM during the first year of follow-up. Continuous variables were tested for normality using the Shapiro–Wilk test. Normally distributed data are expressed as mean ± standard deviation (SD) and were compared using the independent-samples t-test, whereas non-normally distributed data are expressed as median (interquartile range, IQR) and were compared using the Mann–Whitney U test. Categorical variables are expressed as counts and percentages and were analyzed using the Chi-square or Fisher’s exact test, as appropriate.

Since MiR expression values exhibited skewed distributions, log-transformation was applied to approximate a normal distribution. The normality of log-transformed values was also confirmed using the Shapiro–Wilk test. The change in expression between baseline and 3-month follow-up (Δ_3_log miR) was calculated for each miR and analyzed as an independent parameter [LogmiR (3 months post-PM implantation) – LogmiR (immediately post-PM implantation)], which is equivalent to the logarithm of the fold-change log (3 months/baseline). Similarly, changes in echocardiographic parameters, including ΔLVEF [LVEF (immediately post-PM implantation) – LVEF (3 months or 1 year post-PM implantation)] and ΔLV-GLS [LV-GLS (immediately post-PM implantation) – LV-GLS (3 months or 1 year post-PM implantation)], were calculated and used for correlation and regression analyses. Univariate and multivariate logistic regression analysis was conducted to identify prognostic factors for PiCM development. Given the limited number of PiCM events, all multivariable models were intentionally restricted to a maximum of 2–3 possible confounders to avoid overfitting, in accordance with established event-per-variable recommendations. Correlations between Δ_3_logmiR expression levels and echocardiographic parameters were evaluated using Spearman’s rank correlation coefficients. Receiver operating characteristic (ROC) curves were constructed to assess the discriminative ability of significant Δ_3_logmiR parameters and ΔLV-GLS for predicting PiCM (area under the curve [AUC] with 95% confidence intervals [Cis]; optimal cut-off values determined by the Youden index). Pairwise comparisons of AUCs were performed using DeLong’s method.

All statistical analyses were performed using IBM SPSS Statistics, version 25 (IBM Corp., Armonk, NY, USA) and MedCalc, version 22 (MedCalc Software Ltd., Ostend, Belgium). A two-tailed *p* value < 0.05 was considered statistically significant.

## 3. Results

One hundred twenty-six patients with preserved baseline LVEF (>50%) who underwent PM implantation were included in the analysis and followed for 1 year to assess the development of PiCM. The median age was 79.0 years (IQR 73.0–86.0), and 61.9% were males. Median RVP burden was 90% (IQR 85–95) and the mean baseline LVEF was 60.0 ± 4.7%. The observed incidence of PiCM during one-year follow-up was 11.1% (*n* = 14).

Baseline and follow-up characteristics stratified by PiCM development are summarized in Table 1. No significant differences were observed between those with and without PiCM development with respect to age, sex or comorbidities. A significantly higher median RVP burden was observed in those who developed PiCM compared with those who did not (97% vs. 90%, *p* = 0.006). At baseline, patients who developed PiCM had a higher mean LVEF compared with non-PiCM patients (63.4% vs. 58.0%; *p* < 0.001). No significant difference In Δ_3_LVEF was observed between the two groups at 3 months (*p* = 0.654), and no cases of PiCM were identified at that time. However, by 12 months of follow-up, patients who developed PiCM exhibited a significantly greater decline in systolic function (median Δ_3_LVEF 10.0 vs. 2.0; *p* < 0.001). The mean 1-year post PM-implantation LVEF had decreased to 52.3% in the PiCM group compared with 55.8% in non-PiCM patients (*p* = 0.001).

**Figure 1:**
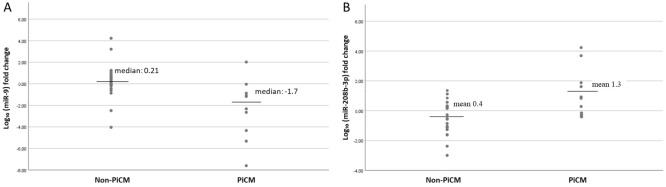
Log10 fold change in miRs expression of (**A**) miR-9 and (**B**) miR208b-3p, stratified by PiCM.

In contrast to LVEF, a significant difference in LV-GLS was already evident at 3 month and remained at 1 year of follow-up. Patients who developed PiCM showed an earlier and more pronounced impairment in longitudinal systolic function, with a median Δ_3_LV-GLS of −2.0 compared with 0.0 in the non-PiCM group (*p* < 0.001). At 1-year follow-up, patients who eventually developed PiCM showed a markedly greater deterioration in LV-GLS compared with non- PiCM patients (−3.0 vs. 0.0, *p* < 0.001). At the molecular level, miR expression levels exhibited distinct and inverse temporal patterns between groups. No statistically significant differences in log (miR) expression levels were observed immediately or 3 months post-PM implantation between patients with and without PiCM. However, the magnitude of change over time was the key differentiating factor, as the PiCM group exhibited a lower median Δ^3^log miR-9 (−1.7 vs. 0.21, *p* = 0.011) and a higher mean Δ_3_log miR-208b (1.3 vs. −0.4, *p* = 0.013) (Figure 1A,B). In an exploratory sex-stratified analysis within the PiCM subgroup, no significant differences were observed between men and women in Δ_3_log miR-9 (*p* = 0.479) or Δ_3_log miR-208b-3p (*p* = 0.865).

At baseline, most patients were minimally symptomatic, with the majority classified as NYHA class I. Over the one-year of follow-up, patients who developed PiCM showed a significant deterioration in functional status, with worsening by one NYHA class compared with baseline (*p* = 0.003). In addition, HF hospitalization occurred more frequently in the PiCM group than in non-PiCM patients (28.6% vs. 6.3%, *p* < 0.001). Notable, no deaths or major arrhythmic events were observed in either group during the 1-year-follow-up period.

Univariate logistic regression analysis (Table 2) was conducted to identify potential factors associated with the development of PiCM. In this study, conventional demographic parameters such as age (*p* = 0.790), sex (*p* = 0.340), and RVP burden (*p* = 0.663) were not associated with the development of PiCM. In contrast, less negative baseline LV-GLS (OR 0.64, *p* = 0.023), and greater changes in Δ_3_LVEF (OR 1.44, *p* = 0.009) and Δ_3_LV-GLS (OR 0.04, *p* = 0.001) were strongly associated with PiCM occurrence. At the molecular level, Δ_3_log miR-208b-3p (OR 2.88, *p* = 0.021) was positively associated with the risk of developing PiCM, while Δ_3_log miR-9 (OR 0.53, *p* = 0.016) appeared to have a protective effect.

To further evaluate whether Δ_3_GLS and Δ_3_logmiR remained independent predictors of PiCM development after adjusting for potential confounders, multivariable logistic regression models were constructed (Table 3). The Δ_3_GLS was an independent predictor of PiCM development in a multivariable model adjusted for age, sex and baseline LVEF (OR 0.002, *p* = 0.048). In addition, both Δ_3_log miR-9 (OR 0.41, *p* = 0.045) and Δ_3_log miR-208b-3p (OR 2.321, *p* = 0.047) emerged as well independent predictors of PiCM in separate multivariable models adjusted for age, sex, and RVP.

**Figure 2:**
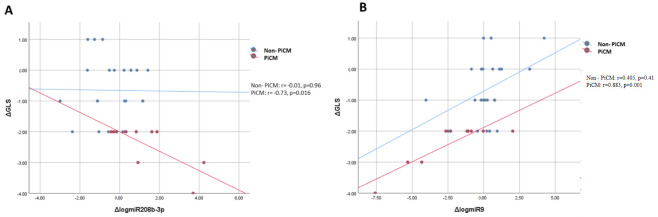
Correlation between Δ_3_LV-GLS and Δ_3_logmiR expression for (**A**) Δ_3_logmiR-208b-3p and (**B**) Δ_3_logmiR-9, stratified by PICM.

Correlations between Δ_3_LV-GLS and Δ_3_log miRs were assessed, as both parameters were significant in multivariate analyses. Among patients who developed PiCM, Δ_3_log miR-208b-3p correlated strongly and inversely with Δ_3_LV-GLS (r = −0.73, *p* = 0.016), indicating that a greater increase in miR-208b-3p expression was associated with more pronounced deterioration in systolic function. Conversely, dynamic changes in miR-9 expression (Δ_3_miR-9) showed a strong positive correlation with Δ_3_LV-GLS (r = 0.88, *p* < 0.001), suggesting that an upregulation of miR-9 over time was linked to better preservation of LV longitudinal function. No significant correlations were observed in non-PiCM patients (r = −0.001, *p* = 0.96; and r = 0.405, *p* = 0.41, respectively). These findings are graphically illustrated in Figure 2A,B. No significant correlations were identified between Δ_3_miR-208b-3p or Δ_3_miR-9 levels and the Δ_3_LVEF in either subgroup (all *p* > 0.26).

**Figure 3:**
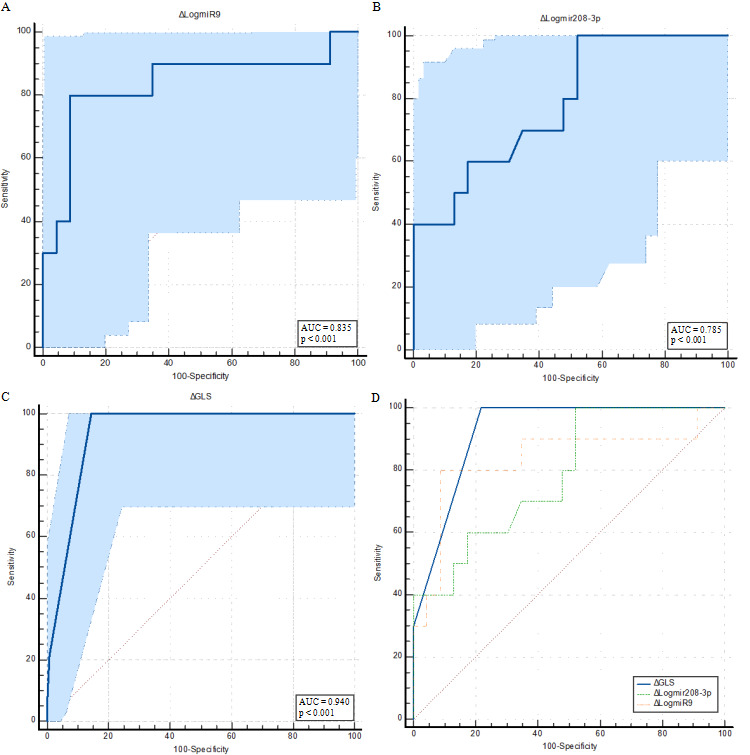
ROC curves of Δ*_3_*log10-miR expression changes and Δ*_3_*LV-GLS for predicting PiCM development: (**A**) Δ*_3_*log_10_(miR-9), (**B**) Δ*_3_*log_10_(miR-208b-3p), and (**C**) Δ*_3_*LV-GLS. (**D**) Comparison of ROC curves for Δ*_3_*log_10_(miR-208b-3p), Δ*_3_*log_10_(miR-9), and Δ*_3_*LV-GLS in predicting PiCM development.

Since both Δ_3_log miR-208b and Δ_3_log miR-9 were significantly correlated with Δ_3_LV-GLS among PiCM patients, ROC curve analyses were subsequently performed to assess their predictive ability for PiCM and to compare them with Δ_3_LV-GLS as a reference functional marker (Figure 3). The area under the curve (AUC) for Δ_3_LV-GLS was 0.924 (95% CI 0.776–0.987; *p* = 0.001). The optimal cut-off value determined by Youden’s J statistic was Δ_3_LV-GLS = 1.5, yielding a sensitivity of 100% and a specificity of 78.3%. For Δ_3_log miR-208b-3p, the AUC was 0.783 (95% CI 0.605–0.907; *p* < 0.001), with an optimal cut-off of 0.698 corresponding to a sensitivity of 60% and specificity of 82.6%. Additionally, the ROC analysis for Δ_3_log miR-9 demonstrated an AUC of 0.835 (95% CI 0.565–0.970; *p* = 0.0004), with an optimal cut-off value of −0.88 providing a sensitivity of 80.0% and specificity of 91.3%. Comparative analysis of the ROC curves was performed using DeLong’s test (Figure 3D). In pairwise DeLong comparisons, although Δ_3_LV-GLS had the higher AUC, none of the pairwise differences were statistically significant (all *p* > 0.05).

## 4. Discussion

PiCM represents an increasingly recognized form of acquired cardiomyopathy in patients with chronic RVP, particularly among those who are highly PM-dependent. In clinical practice, early detection of subclinical systolic impairment remains a key challenge, as conventional parameters such as LVEF often decline only after substantial myocardial remodeling has occurred. This study is one of the few to explore the role of miR expression levels in PBMCs as potential predictive biomarkers for the development of PiCM.

Principal findings of our study are: [1] the observed 1-year incidence of PiCM was 11.1% [2]. At 3 months, patients who developed PiCM exhibited a more pronounced decrease in LV-GLS and opposing regulation of miR expression levels—an upregulation of miR-208b-3p and a downregulation of miR-9—compared with non-PiCM patients. In univariate analysis, these factors were found to be associated with the development of PiCM. Multivariable analyses showed that a more negative Δ_3_GLS (OR = 0.002, *p* = 0.048), a lower Δ_3_log miR-9 (OR = 0.410, *p* = 0.045), and a higher Δ_3_log miR-208b-3p (OR = 2.321, *p* = 0.046) were independent predictors of PiCM development [3]. Among PiCM patients, dynamic changes in miR-208b-3p and miR-9 expression correlated inversely and positively, respectively, with changes in LV-GLS, whereas no significant correlations were observed between changes in miR expression and LVEF [4]. In ROC curve analysis, changes in miR expression levels (Δ_3_logmiR-208b-3p, Δ_3_logmiR-9) demonstrated predictive performance comparable to Δ_3_LV-GLS, supporting their role as potential complementary biomarkers for the early identification of PiCM.

The observed finding that early decline in LV-GLS in those who eventually developed PiCM is in line with previous studies [20,21]. Chronic right ventricular apical pacing induces a left bundle branch block (LBBB)-type activation pattern, leading to delayed and asynchronous LV electrical activation and mechanical contraction [22]. This dyssynchrony predominantly affects the subendocardial longitudinal fibers, which are highly dependent on coordinated activation. As a result, a reduction in LV-GLS is observed, reflecting impaired LV systolic performance even before a measurable decline in ejection fraction becomes apparent [23,24]. In addition, both baseline LV-GLS and its subsequent 3-month change were significantly associated with the development of PiCM, with each 1% reduction in baseline LV-GLS corresponding to approximately 36% higher odds of developing PiCM. When Δ_3_GLS was evaluated in the multivariable models, its association with PiCM remained significant even after adjustment for age, sex, and baseline LVEF. It is noteworthy that a higher baseline LVEF also emerged as an independent predictor. This finding should be interpreted with caution, as all patients entered the study with preserved LVEF by design, resulting in a restricted range and a ceiling effect that may artificially exaggerate the contribution of baseline LVEF. Furthermore, patients with higher baseline LVEF may have had subtle diastolic abnormalities or impaired ventricular compliance that are not captured by LVEF alone and could predispose them to pacing-related functional decline. Nevertheless, despite the inclusion of baseline LVEF, which was expected to compete strongly with Δ_3_GLS due to its opposite directionality, and the limited number of PiCM events that increases coefficient instability, Δ_3_GLS remained independently predictive. This supports the interpretation that early impairment in myocardial deformation reflects a genuine susceptibility to pacing-induced dysfunction rather than a secondary artifact of baseline LVEF differences.

To the best of our knowledge, this is the first study to explore the association between dynamic changes in miR-208b-3p and miR-9 expression levels and PiCM in patients with high RVP burden and preserved baseline LVEF. Current literature in molecular cardiology allows us to assert that miRs might represent important regulators of cardiac injury and remodeling, serving as potential early diagnostic or prognostic biomarkers of subclinical myocardial dysfunction [25,26,27]. According to our findings, within three months, patients who subsequently developed PiCM exhibited significant upregulation of miR-208b-3p and downregulation of miR-9 compared to those who did not. After adjusting for age, sex, and RVP burden, greater Δ_3_logmiR-208b-3p and lower Δ_3_logmiR-9 remained associated with an increased likelihood of developing PiCM. Although biologically plausible, these findings should be interpreted in light of the small number of events. Among the currently identified miRs, miR-208 is uniquely expressed in myocardial tissue and has been implicated in hypertrophic and fibrotic signaling, as well as conduction remodeling, being elevated in myocardial injury, arrhythmia and HF [28,29,30]. Specifically, miR-208 regulates myosin heavy chain isoform expression—particularly β-MHC—and influences fibrotic signaling cascades such as the TGF-β/Smad pathway [31]. Zhang et al., observed that miR-208b promotes cardiac fibrosis via activation of TGF-β1/Smad3 signaling, highlighting its regulatory role in fibroblast activation and extracellular matrix deposition [32]. Moreover, miR-208 contributes to electrical remodeling by modulating connexins (Cx40/Cx43) and the miR-208/Mef2 axis in hypertrophic myocardium [33]. In experimental models, deletion of miR-208 blunts hypertrophic growth and attenuates fibrosis under pressure overload [34].

Conversely, available data indicate that miR-9 expression is as well altered during cardiac remodeling, reflecting its involvement in distinct pathophysiological mechanisms. One plausible pathophysiological explanation is that MiR-9 exerts anti-fibrotic effects by targeting TGFBR2 and NOX4 and inhibiting fibroblast-to myofibroblast transition [35,36], supporting a protective regulator against pathological cardiac remodeling. According to a study regarding diabetic cardiomyopathy, miR-9 downregulation under hyperglycemia promoted fibroblast activation and collagen accumulation through TGF-β/Smad3 signaling [37]. Experimental overexpression of miR-9 in rat myocardium reduces fibrosis and Smad2/3 phosphorylation [38], while mesenchymal stem cell-derived exosomes enriched in miR-9-5p have been shown to attenuate cardiac fibrosis and improve ventricular performance in injury models [39]. Beyond the heart, miR-9-5p antagonizes TGF-β-driven fibrogenic signaling in pulmonary and hepatic fibroblasts [35]. A second pathophysiological mechanism involves anti-inflammatory actions. MiR-9 suppresses key inflammatory pathways, including NF-κB activation [40,41] and downstream cytokine release (TNF-α, IL-1β, IL-6) [42]. A retrospective cohort study including 41 COVID-19 patients observed that miR-9 levels were higher in individuals with COVID-19, particularly in non-survivors, and were strongly correlated with increased levels of IL-6, IL-1β, and TNF-α [43]. Recently, D’Amato et al., demonstrated that miR-9 also regulates immune signaling by promoting macrophage polarization toward the pro-inflammatory M1 phenotype and modulating local inflammatory pathways—processes that may exacerbate remodeling and progression toward heart failure [44]. Beyond its established anti-inflammatory and anti-fibrotic roles, miR-9 also participates in structural remodeling through a distinct mechanistic axis involving endothelial–fibroblast signaling. In non-diabetic cardiac injury, endothelial and fibroblast miR-9 activity, regulated through interactions with lncRNAs such as ZFAS1, has been linked to extracellular matrix remodeling and fibrotic progression [13]. Another explanation involves its anti-hypertrophic actions, since experimental data demonstrate that it directly suppresses myocardin and NFATc3, two central transcriptional regulators of pathological hypertrophy. Downregulation of miR-9 leads to activation of hypertrophic gene programs (ANP, BNP, β-MHC) and subsequent maladaptive remodeling. In contrast, literature also reports conditions in which miR-9 levels increase in response to cardiac injury. In acute ischemic myocardial infarction models, miR-9-5p is upregulated under hypoxic stress, reduces fibrosis, and limits cardiomyocyte death. This indicates that in the setting of acute ischemia, the miR-9-5p/FSTL1 axis may be maladaptive [45]. However, PiCM arises through a distinct, non-ischemic mechanism driven by chronic pacing-induced dyssynchrony and mechanical overload rather than acute hypoxic injury. Consequently, the decrease in Δ_3_logmiR-9 observed in our study is more consistent with findings from pressure-overload and other mechanical-stress models, in which miR-9 suppression facilitates hypertrophic, inflammatory, and fibrotic remodeling.

The correlations observed in our study are consistent with the above-mentioned findings and suggest a potential link between molecular and functional remodeling in PiCM. Upregulation of Δ_3_logmiR-208b-3p expression correlated inversely with the change in Δ_3_LV-GLS, whereas downregulation of Δ_3_logmiR-9 showed a positive correlation, indicating that molecular alterations may parallel early deterioration in left ventricular mechanics. It is worth noting that only a few studies, mostly in patients with aortic stenosis or coronary artery disease, have reported associations between LV-GLS and miRs such as miR-21, miR-29, or miR-133, linking them to fibrotic or hypertrophic remodeling processes [46,47,48]. Building upon our observations, ROC curve analysis of the three parameters demonstrated good discriminatory performance, with miR expression levels showing predictive ability comparable to that of ΔLV-GLS. Considering that previous studies have demonstrated early LV-GLS deterioration to be a sensitive marker of subclinical myocardial fibrosis and a reliable predictor of PiCM development [49,50], the comparable predictive ability observed for Δ_3_logmiR-208b-3p and Δ_3_logmiR-9 in our study suggests that molecular biomarkers could be used either independently or in conjunction with LV-GLS to enhance early risk stratification. Given this, a combined model incorporating both echocardiographic parameters such as LV-GLS and molecular markers such as miR-208b-3p and miR-9 is conceptually attractive, as these modalities capture complementary aspects of PiCM. However, the present study was not powered to evaluate incremental predictive value, and constructing such a combined model with only 14 PiCM events would yield unstable estimates. Therefore, formal incremental analysis and validation of cutoff values will require larger independent cohorts before clinical implementation is considered.

Mechanistically, pacing-induced dyssynchrony likely initiates cellular stress responses that promote both mechanical strain and biochemical signaling changes in cardiomyocytes and fibroblasts. The selective release of miRs such as miR-208b-3p and miR-9 into circulation may represent a response to mechanical overload or early cellular injury [14,51,52]. Whether these miRs exert paracrine or endocrine effects remains uncertain; however, emerging evidence indicates that miRs expressed within PBMCs can influence cardiac remodeling indirectly through immune-mediated mechanisms [53]. PBMC-derived miRs may modulate cytokine production, inflammatory activation, and fibroblast–immune cell crosstalk, thereby shaping the myocardial microenvironment during early remodeling [54]. Activated PBMCs may also release exosomes and microvesicles enriched with regulatory miRs, which can be taken up by cardiomyocytes, fibroblasts, and endothelial cells [55]. While these intracellular pathways have been described in other cardiovascular contexts, it remains unknown whether the specific miRs identified in our study (miR-208b-3p, miR-9) participate in such signaling in the context of pacing-induced stress or whether their changes primarily reflect PBMC transcriptional responses to early dyssynchrony-related remodeling.

Furthermore, it remains unclear which method is most effective for the reliable detection of cardiac miRs. Measuring miRs in plasma or serum has clear practical advantages for clinical translation, but also greater susceptibility to confounders such as hemolysis, exosome-release variation and dilution from plasma volume changes [56,57]. Even though PBMC isolation is more complex and time-consuming, this method was employed in our study to minimize variability from these sources and to focus on an intracellular compartment likely to reflect mechanistic changes rather than solely circulating signals. According to the existing literature, the choice of biological matrix depends on the underlying pathophysiology. PBMC-derived miRNAs are more commonly used in chronic cardiovascular conditions (CHF, stable CAD, dilated cardiomyopathy) where they appear to reflect sustained inflammatory and fibrotic signaling within immune cells [17,18]. Conversely, circulating miRNAs are typically used in acute myocardial injury (e.g., myocardial infarction, acute myocarditis), where rapid cardiomyocyte release produces strong early plasma signals [12,58,59]. Therefore, although plasma miRNAs are more practical for clinical use, PBMC-derived miRNAs may be better suited for detecting the gradual and subclinical remodeling characteristic of PiCM.

Experimental gain-and loss-of-function models have shown that several cardiac miRs, such as miR-208 and miR-9 can directly participate in hypertrophic, fibrotic and inflammatory signaling pathways, suggesting a potential mechanistic role in adverse remodeling. Nevertheless, human clinical studies have largely positioned circulating and cellular miRs as biomarkers of myocardial stress rather than demonstrated causal mediators [60,61]. Clarifying whether the miRs changes a cause of myocardial remodeling, a consequence of early subclinical dysfunction, or simply biomarkers reflecting parallel processes will require studies with more frequent sampling, mechanistic interventions and integrated omics approaches. In this study, baseline miR levels did not differ between patients who later developed PiCM and those who did not, suggesting that fixed pre-existing molecular predisposition was not detectable. Instead, the dynamic changes may represent an early response to dyssynchrony-related mechanical stress or subclinical myocardial injury, and thus could be effectively utilized as predictive biomarkers.

In our cohort, approximately one in four patients who developed PiCM required hospitalization for HF decompensation within the first year. This is consistent with the observed worsening in functional status, as most affected patients experienced a decline of one NYHA class compared with baseline. The clinical relevance of our findings is supported by prior studies evaluating outcomes in patients who develop PiCM. In a large cohort of 618 patients, those who developed PiCM exhibited a markedly higher rate of adverse events, with an almost threefold increased risk of HF hospitalization compared with individuals without PiCM [62]. Unlike that 5-year follow-up study, which reported an increased risk of all-cause mortality, no deaths occurred during the one-year follow-up in our cohort, an expected difference given the considerable shorter observation period. Similarly, in a study of 170 patients undergoing chronic RVP, a trend toward a higher prevalence of advanced symptoms was observed among individuals who eventually developed PiCM, with NYHA class III–IV occurring more frequently over 12 months [63].

This study has certain limitations. The main limitation is the relatively low number of PiCM events, which restricts statistical power and limits the power of our multivariable analysis. To mitigate the risk of overfitting, adjusted models were restricted to a maximum of three covariates; however, given the event-per-variable ratio, these findings should be interpreted as exploratory. Although Δ_3_LV-GLS, Δ_3_log miR-9 and Δ_3_log miR-208b-3p were associated with PiCM development in adjusted analyses, the limited number of events precludes definitive conclusions regarding their independent predictive value. Another limitation is that we did not obtain plasma samples in parallel and therefore could not directly compare PBMC-derived microRNAs with circulating levels, underscoring the need for future studies to include paired plasma/serum and PBMC sampling to assess concordance and determine which matrix offers the best balance between practicality and biological relevance. In addition, the observational, single-center design may introduce selection bias. The observed incidence reflects our cohort but may not generalize to broader populations. Nevertheless, the University General Hospital of Heraklion serves as a tertiary referral and specialized pacing center, providing care for a large and heterogeneous patient population across Crete and the southern Aegean region. Larger prospective, adequately powered multicenter studies with broader miR profiling and longer follow-up are warranted to validate these findings and further elucidate the diagnostic and prognostic role of miR-208b-3p and miR-9 expression levels in PiCM. Despite these limitations, the study has several strengths. The study population, although modest in size, is representative of the real-world cohort of PM recipients with preserved LVEF, in whom the incidence of PiCM is relatively low. The inclusion of a control group without PiCM enabled direct comparison of molecular and functional parameters, increasing the robustness of the observed associations. Furthermore, the assessment of both echocardiographic and molecular markers at an early time point (3 months and 1 year) provided novel insights into the temporal sequence of PiCM.

## 5. Conclusions

In conclusion, in our study, patients who ultimately developed PiCM after 1 year of follow-up demonstrated early LV-GLS deterioration, upregulation of miR-208b-3p and downregulation of miR-9 in PBMCs within 3 months post PM-implantation, changes which preceded subsequent reductions in LVEF. In adjusted analyses, Δ_3_LV-GLS, Δ_3_logmiR-208b-3p and Δ_3_logmiR-9 were associated with a higher likelihood of PiCM. However, these results should be interpreted as exploratory given the limited number of PiCM events. Larger prospective, multicenter studies are warranted to confirm whether ΜiRs expression could complement LV-GLS for the timely identification of patients at risk of PiCM and to establish their prognostic utility.

## 6. Clinical Implications

Several potential implications for clinical practice can be derived from the findings of this study. First, our results show that early dynamic changes in miR-208b-3p and miR-9 expression levels in PBMCs provide predictive information comparable to early deterioration in LV-GLS. This suggests that molecular biomarkers could complement or potentially enhance current echocardiographic surveillance strategies in patients with high RVP. Because conventional parameters such as LVEF typically decline only after extensive myocardial remodeling, the use of miRNAs and LV-GLS may allow clinicians to identify patients at risk of PiCM at an earlier stage, when interventions are more likely to be effective.

## 7. Future Directions

Further research is needed to validate and extend these observations. First, larger multicenter studies with adequate power are necessary to confirm the predictive performance of miR-208b-3p and miR-9 and to establish optimal cutoff values for clinical use. Second, pairing PBMC-derived miRNA measurements with circulating plasma or serum miRNA levels will help determine which biological matrix offers the best combination of practicality and mechanistic relevance for real-world implementation.

Additionally, expanded molecular profiling—including other fibrosis-, inflammation-, and hypertrophy-related miRNAs—may reveal broader signatures of pacing-induced remodeling. Mechanistic studies are also warranted to clarify whether PBMC-derived miRNAs actively participate in myocardial remodeling through immune–cardiac signaling pathways or simply reflect systemic responses to pacing-induced stress. Finally, longitudinal studies with longer follow-up will be essential to assess whether early biomarker changes translate into meaningful differences in long-term clinical outcomes and whether early intervention strategies based on these markers improve prognosis.

## Figures and Tables

**Table 1:** Baseline and follow-up characteristics stratified by PiCM development.

Variables	Total Population (n = 126)	PiCM (n = 14)	Non PiCM (n = 112)	*p*-Value
Age, median (IQR)	79.0 (73.0, 86.0)	80.5 (76.8, 82.5)	79.0 (72.0, 86.8)	0.849
Males, n (%)	78 (61.9)	7 (9.0)	71 (91.0)	0.390
Smoking, n (%)	18 (14.3)	1 (5.6)	17 (94.4)	0.690
AH, n (%)	84 (66.7)	11 (13.1)	73 (86.9)	0.383
T2D, n (%)	61 (48.4)	4 (6.6)	57 (93.4)	0.158
Hyperlipidemia, n (%)	70 (55.6)	9 (12.9)	61 (87.1)	0.576
CKD, n (%)	61 (48.8)	5 (8.2)	56 (91.8)	0.398
History of HF, n (%)	32 (25.4)	2 (6.3)	30 (93.8)	0.516
History of AF, n (%)	27 (21.4)	6 (22.2)	21 (77.8)	0.076
History of CAD, n (%)	17 (13.5)	1 (5.9)	16 (94.1)	0.691
RVP%, median (IQR)	90.0 (85.0, 95.0)	97.0 (89.5, 99.0)	90.0 (85.0, 95.0)	**0.006**
Baseline BNP, median (IQR)	149.0 (66.0, 430.0)	131.5 (60.8, 360.0)	149.0 (66.0, 436.0)	0.521
Baseline LVESD, mean (SD)	35.0 (±3.8)	35.1 (±4.5)	36.1 (±3.8)	0.455
Baseline LVEDD, median (IQR)	48.0 (46.0, 50.0)	48.5 (46.3, 53.5)	48.0 (46.0, 49.8)	0.297
Baseline LVEF, mean (SD)	60.0 (±4.7)	63.4 (±2.4)	58.0 (±4.6)	**<0.001**
Δ_3_LVEF, median (IQR)	1.0 (−2.0, 2.5)	2.0 (1.0, 3.0)	1.0 (−2.0, 2.0)	0.065
Δ 1-year LVEF, median (IQR)	3.0 (0.5, 5.3)	10.0 (10.0, 11.0)	2.0 (−1.0, 5.0)	**<0.001**
1-year LVEF, mean (SD)	55.4 (±4.5)	52.3 (±2.8)	55.8 (±4.5)	**0.001**
Baseline LV-GLS, median (IQR)	−16.0 (−17.0, −16.0)	−17.0 (−19.0, −16.0)	−16.0 (−17.0, −16.0)	0.136
Δ_3_LV-GLS, median (IQR)	0.0 (−1.0, 0)	−2.0 (−2.25, −2.0)	0.0 (−1.0, 0)	**<0.001**
Δ 1-year LV-GLS, median (IQR)	0.0 (−1.3, 0)	−3.0 (−4.0, −2.0)	0.0 (−1.0, 0)	**<0.001**
Δ_3_log(miR-9), median (IQR)	−0.04 (−1.1, 0.7)	−1.7 (−4.6, −0.6)	0.21 (−0.2, 0.76)	**0.011**
Δ_3_log(miR-280b-3p), mean (SD)	0.22 (±1.5)	1.3 (±1.6)	−0.4 (±1.2)	**0.013**
Baseline NYHA score, median (IQR)	1.0 (1.0, 2.0)	1.0 (1.0, 1.0)	1.0 (1.0, 2.0)	0.290
1-year NYHA score, median (IQR)	1.0 (1.0, 2.0)	2.0 (1.0, 2.0)	1.0 (1.0, 2.0)	**0.030**
1-year Hospitalization, n (%)	14 (8.7)	4 (28.6)	7 (6.3)	**0.020**

AF, atrial fibrillation; AH, hypertension; BNP, B-type natriuretic peptide; CAD, coronary artery disease; CKD, chronic kidney disease; ECG, electrocardiogram; HF, heart failure; LV-GLS, left ventricular global longitudinal strain; LVEF, left ventricular ejection fraction; LVEDD, left ventricular end-diastolic diameter; LVESD, left ventricular end-systolic dimension; NYHA, New York Heart Association Functional Classification; PiCM, pacing-induced cardiomyopathy; RVP, right ventricular pacing; T2D, type 2 diabetes.

**Table 2:** Univariate analysis of prognostic factors for PiCM development.

Univariate Analysis	Exp(B)	95% CI	*p*-Value
age	1.008	0.951, 1.068	0.790
Male sex	0.577	0.189, 1.763	0.340
RVP	1.018	0.938, 1.105	0.663
Baseline LVEF	1.558	1.215,1.999	**<0.001**
Baseline LV-GLS	0.642	0.438, 0.940	**0.023**
Δ_3_LVEF	1.443	1.095,1.903	**0.009**
Δ_3_LV-GLS	0.041	0.006, 0.281	**0.001**
Δ_3_logmiR-280b-3p	2.882	1.177, 7.057	**0.021**
Δ_3_logmiR-9	0.527	0.313, 0.889	**0.016**

LV-GLS, left ventricular global longitudinal strain; LVEF, left ventricular ejection fraction; PiCM, pacing-induced cardiomyopathy; RVP, right ventricular pacing.

**Table 3:** Multivariate analysis of prognostic factors for PiCM development.

Model	Predictor	OR (95% CI)	*p*-Value
Model 1 A Β C	Δ_3_LV-GLS	0.041 (0.006–0.282)	**<0.001**
	Δ_3_LV-GLS	0.040 (0.006–0.274)	**0.001**
	Δ_3_LV-GLS	0.002 (0.000–1.468)	**0.048**
Model 2 A Β C	Δ_3_log miR-9	0.605 (0.396–0.924)	**0.020**
	Δ_3_log miR-9	0.585 (0.356–0.963)	**0.035**
	Δ_3_log miR-9	0.410 (0.160–1.049)	**0.045**
Model 3 A Β C	Δ_3_log miR-208b	2.664 (1.125–6.312)	**0.026**
	Δ_3_log miR-208b	2.426 (1.020–5.770)	**0.045**
	Δ_3_log miR-208b	2.321 (1.016–5.305)	**0.047**

Values are given as odds ratio (OR) and 95% confidence interval. Model 1 [1A: adjusted for age. 1B: adjusted for age and sex. 1C: adjusted for age, sex and baseline LVEF]. Model 2 [2A: adjusted for age. 2B: adjusted for age and sex. 2C: adjusted for age, sex and RVP]. Model 3 [3A: adjusted for age. 3B: adjusted for age and sex. 3C: adjusted for age, sex and RVP].

## Data Availability

The data that support the findings of this study are openly available.

## Abbreviations

The following abbreviations are used in this manuscript:

AF	Atrial fibrillation
AH	Arterial hypertension
AUC	Area under the curve
AV	Atrioventricular
BNP	Beta-type natriuretic peptide
CAD	Coronary artery disease
CHD	Congenital heart disease
CRP	C-reactive protein
Cx40/Cx43	Connexin 40/Connexin 43
HF	Heart failure
LBBB	Left bundle branch block
lncRNA	Long non-coding RNA
LV	Left ventricle / Left ventricular
LVEDD	Left ventricular end-diastolic diameter
LVEF	Left ventricular ejection fraction
LVESD	Left ventricular end-systolic diameter
LV-GLS	Left ventricular global longitudinal strain
M1	Macrophage type 1 (pro-inflammatory phenotype)
Mef2	Myocyte enhancer factor 2
miR	MicroRNA
MI	Myocardial infarction
MYH7	β-myosin heavy chain gene
NYHA	New York Heart Association Functional Classification
PBMCs	Peripheral blood mononuclear cells
PiCM	Pacing-induced cardiomyopathy
PM	Pacemaker
PPIs	Proton pump inhibitors
qPCR	Quantitative polymerase chain reaction
RNA	Ribonucleic acid
ROC	Receiver operating characteristic
RVP	Right ventricular pacing
T2D	Type 2 diabetes
TGF-β	Transforming growth factor beta
TGFβRII	Transforming growth factor beta receptor II
VHD	Valvular heart disease
